# Determination of Protein Structural Ensembles by Hybrid-Resolution SAXS Restrained Molecular Dynamics

**DOI:** 10.1021/acs.jctc.9b01181

**Published:** 2020-03-02

**Authors:** Cristina Paissoni, Alexander Jussupow, Carlo Camilloni

**Affiliations:** † Dipartimento di Bioscienze, Università degli Studi di Milano, via Celoria 26, 20133 Milano, Italy; ‡ Department of Chemistry and Institute of Advanced Study, Technical University of Munich, Garching 85747, Germany

## Abstract

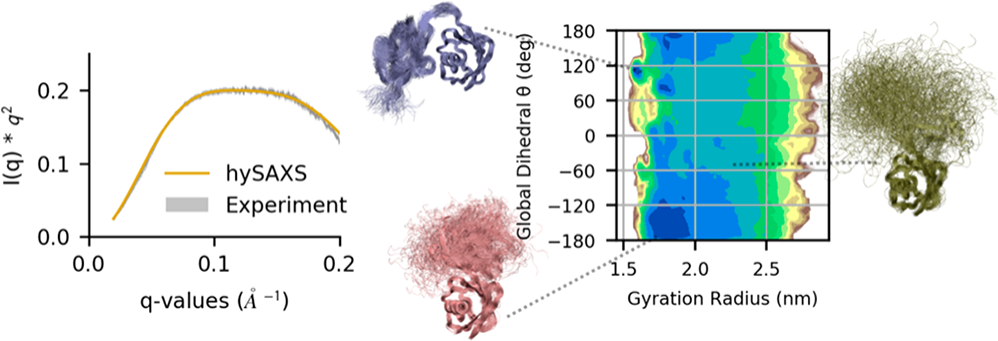

Small-angle X-ray scattering (SAXS) experiments provide low-resolution but valuable information about the dynamics of biomolecular systems, which could be ideally integrated into molecular dynamics (MD) simulations to accurately determine conformational ensembles of flexible proteins. The applicability of this strategy is hampered by the high computational cost required to calculate scattering intensities from three-dimensional structures. We previously presented a hybrid resolution method that makes atomistic SAXS-restrained MD simulation feasible by adopting a coarse-grained approach to efficiently back-calculate scattering intensities; here, we extend this technique, applying it in the framework of metainference with the aim to investigate the dynamical behavior of flexible biomolecules. The efficacy of the method is assessed on the K63-diubiquitin, showing that the inclusion of SAXS restraints is effective in generating a reliable conformational ensemble, improving the agreement with independent experimental data.

## 1 Introduction

Biomolecules in solution can be characterized by a different extent of conformational dynamics, depending on the specific system and experimental conditions.^1−3^ While the dynamics of single-domain proteins under native conditions is generally limited to fluctuations around a well-defined structure, fully disordered proteins can only be described as statistical ensembles of conformations. Between these cases, multidomain proteins connected by linker regions can populate multiple states generally characterized by a different size.^4^

Experimentally, the characterization of conformational heterogeneity can be achieved by employing multiple solution techniques, such as nuclear magnetic resonance (NMR), Förster resonance energy transfer (FRET), and small-angle X-ray scattering (SAXS).^1,2^ The latter has the advantages of being label-free, as well as having the ability to work with systems of any size and operate under essentially all experimental conditions.^5^ An atomistic interpretation of scattering data could benefit from its combination with computational techniques, such as molecular dynamics (MD) simulations, which could provide an accurate physical model to generate reliable conformational ensembles, in agreement with SAXS data.^6,7^ Common approaches employ SAXS to reweight conformational ensembles a posteriori, making use of statistically founded theoretical frameworks.^8−14^ Recently, few methods in which SAXS experimental data are integrated into MD to drive conformational sampling have been proposed; nevertheless, their application is hindered by the high computational cost required to calculate scattering intensities.^15−18^

In a previous work,^19^ we developed a MD-based multiresolution strategy to efficiently refine protein-DNA and protein-RNA complexes integrating SAXS experimental data with metainference.^20^ According to this strategy, MD is performed with full atomistic details, using standard atomistic force fields, while the back-calculation of SAXS intensities is performed in a coarse-grain fashion,^21^ based on the Martini force field.^22^ In the refinement protocol, conformational averaging was not considered, under the assumption that a single structure, representing the most populated state of the system, could reliably reproduce all of the measured experimental data used as restraints.

In this work, we aim to further extend our MD-based multiresolution approach for the integration of SAXS data in the case of biomolecules that can adopt multiple conformations in solution. To this aim, we propose to take advantage of metainference technique,^20^ which allows one to account for conformational flexibility by restraining the average over multiple simulations (i.e., replica) to fit with input experimental data. Importantly, multireplica metainference simulations combined with metadynamics (M&M)^23,24^ have been previously exploited to integrate NMR data, showing that the inclusion of experimental restraints allows one to correct force-field limitations, leading to well-converged conformational ensembles, independent from the employed force field.^25^

**Figure 1:**
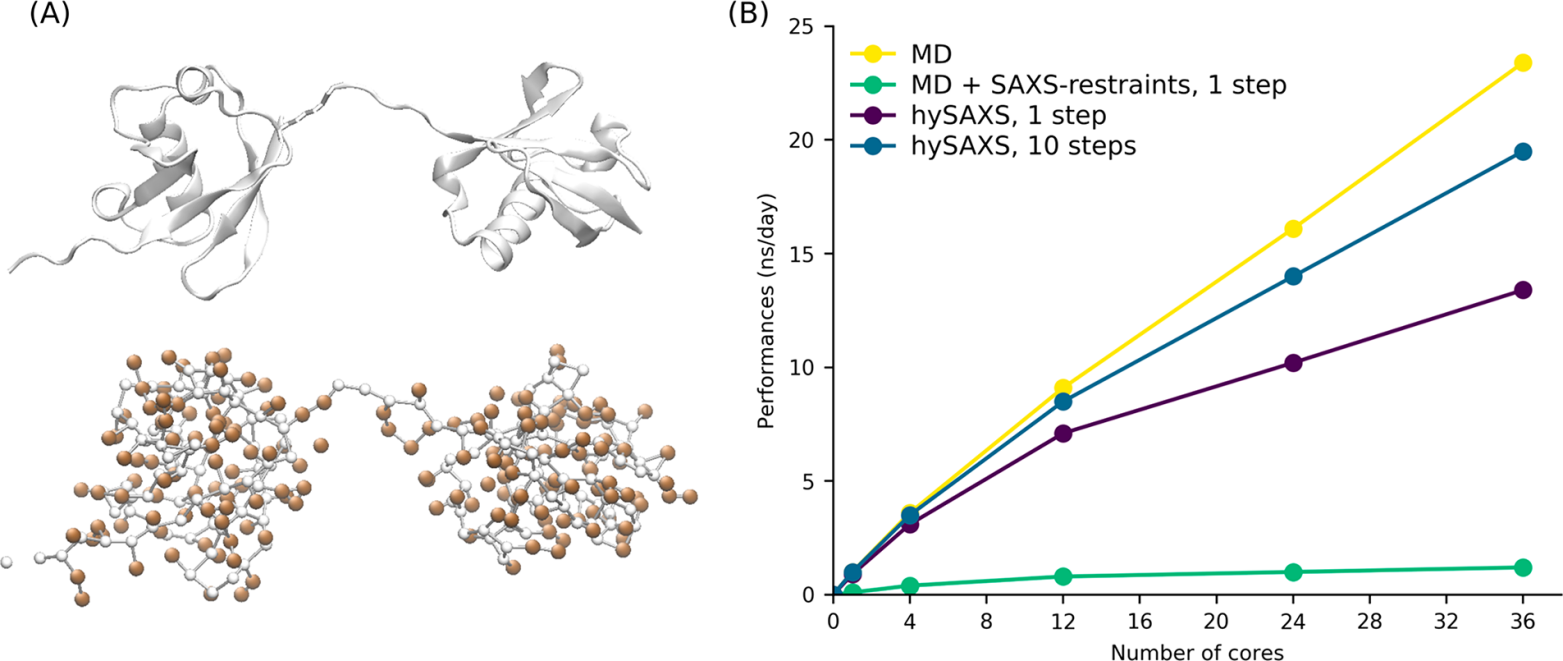
(A) K63-Ub_2_ (2473 atoms), shown as a cartoon representation (top) or highlighting the centers of the 328 Martini beads (bottom), colored in white and orange for backbone and side chain, respectively. Figures were created with VMD software.^66^ (B) Performances, as a function of the number of cores, estimated on Intel Xeon E5-2697 2.30 GHz for a single replica of K63-Ub_2_ in water.

Here, we applied our multiresolution strategy to investigate the conformational ensemble of K63-linked diubiquitin (K63-Ub_2_). Diubiquitins represent an ideal test system, since they are known to populate multiple conformational states, because of the presence of a highly flexible linker connecting the C-terminal of the distal ubiquitin with either a lysine or the N-terminus methionine of the proximal domain (Figure 1A).^26−32^ In particular, the heterogeneity of K63-Ub_2_ conformational space is supported by the presence of numerous crystallographic structures of this protein, free or in complex with diverse targets, displaying different degrees of opening and arrangements of the two subunits.^33−39^ Furthermore, studies based on different biophysical techniques, including SAXS, NMR, cross-linking, and FRET, support the hypothesis that K63-Ub_2_ in solution populates a dynamic ensemble, including both extended and compact states.^31,32,40^ This equilibrium between multiple states is considered critical in modulating the affinity of diubiquitin toward its biological partners.^31^.

In the following, we present our SAXS-restrained all-atom M&M^1,23,41^ simulation of K63-Ub_2_, performed with the hybrid resolution approach (hySAXS simulation), in comparison with an unrestrained reference simulation, in which the same setting was used except for the inclusion of experimental data. Both conformational ensembles indicate an equilibrium between extended and compact conformations, but their assessment with independent experimental NMR paramagnetic relaxation enhancement (PRE) experiments^32^ reveals that only the hySAXS restrained simulations can accurately describe the specific contacts responsible for the formation of compact states. All of the methods described in this paper are freely available in the PLUMED-ISDB module^42^ of the PLUMED library;^43^ furthermore, all of the input files used are available on the PLUMED-NEST repository,^44^ as plumID:19.057.

## 2 Theory and Methods

### 2.1 Metainference

Metainference, combining Bayesian inference and replica-averaging modeling, allows one to integrate experimental data with prior information (generally represented by a molecular mechanic force field), taking into account the effect of conformational averaging.^20^ Following the replica-averaging modeling strategies, multiple replicas of the system are simulated in parallel and the quantities to be restrained against experimental data are back-calculated as an average over the replica. Importantly, the combination of this technique with the statistical basis provided by Bayesian inference allows to tune the strength of the restraints dealing with diverse sources of errors, including random and systematic errors, as well as inaccuracies of the forward model. This is particularly important when using SAXS intensities as restraints, to account for both the noise in the data and for the possible approximations of the forward model (e.g., the coarse-grain representation and the lack of hydration layer). In the case of Gaussian noise, the metainference energy, representing the optimal balance between force field energy (*E*_FF_) and experimental data, can be written as^25^
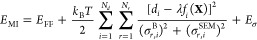
where *k*_B_ is the Boltzmann constant, *T* the temperature, and *d*_*i*_ the set of *N*_d_ experimental data. The term *f_i_*(**X**), which is given as *f_i_*(**X**) = , is averaged over the *N*_*r*_ replicas; *f*_*i*_(*X*_*r*_) is the forward model used to predict observable *i* from conformation *X*_*r*_, σ_*r*,*i*_^Bias^ is an uncertainty parameter that describes random and systematic errors, σ_*r*,*i*_^SEM^ is the standard error of the mean related to the conformational averaging and *E*_σ_ is an energy term that accounts for normalization of the data likelihood and error priors. Monte Carlo sampling is used to sample the uncertainty σ_*r*,*i*_^Bias^ and, optionally, a scaling parameter λ that relates experimental and back-calculated data (as in the case of SAXS experiment): these parameters are inferred during the simulation, along with the model of the system. Importantly, if only one replica is considered, metainference becomes equivalent to the Inferential Structure Determination approach,^45^ in which Bayesian inference is exploited to combine experimental data (with proper statistical treatment of errors) and physical properties, eventually determining the probability distribution of an unknown structure and its precision. Conversely, if σ_*r*,*i*_^Bias^ = 0, metainference is equivalent to the replica-averaged MaxEnt modeling,^46^ in which errors are defined a priori to be as small as possible, rather than being determined during the simulation, along with the model of the system, thus not guaranteeing a proper statistical treatment of data and forward model errors.

Metainference can be combined with metadynamics to accelerate the exploration of the conformational space.^23,47^ In particular, it was proposed to apply it in combination with parallel bias metadynamics^48^ (PBMetaD), which allows the use of many collective variables (CVs) applying multiple low-dimensional bias potentials and therefore reducing the risk of missing slow degrees of freedom. In M&M, multiple copies of the simulation are run in parallel, where all the replicas use the same conditions and force field and share the bias potential, as in the case of the multiple-walkers method.^49^ The coupling of metainference and metadynamics is given by the calculation of the average forward model *f*_*i*_(**X**), where each replica contributes differently to the average with a weight *w*(*X*_*r*_), depending on the bias potential *V*_PB_, according to *w*(*X*_*r*_) = exp[*V*_PB_(CV(*X*_*r*_),*t*)/(*k*_B_*T*)].

### 2.2 Hybrid-Resolution SAXS-Driven Metainference Simulations

Given a coarse-grain representation of a molecule of *N* atoms as a collection of *M* beads, each comprising a variable number of atoms, if the form factors *F*(*q*) of the beads are known, the scattering intensities can be approximated as
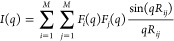
where *R*_*ij*_ indicates the distance between the center of mass of beads *ij* and with the sum running over the number of beads. Therefore, the complexity is reduced from *O*(*N*^2^) to *O*(*M*^2^). The form factors *F*(*q*) for custom beads can be computed by adopting the Single Bead Approximation averaging over multiple structures.^21^ The level of beads coarse-graining is critical to determine the accuracy and the efficiency of the SAXS back-calculation, where large beads representing many atoms are more efficient but less accurate than smaller ones, containing only few atoms. A thoughtful comparison between schemes with different resolution (from atomistic to one-bead per amino-acid) was previously reported.^50^ While one bead per amino acid or coarser schemes could be useful to efficiently simulate large biological system, at the cost of a resolution loss and strongly limiting the range of scattering angles, biomolecular system containing few hundreds of amino acids clearly benefit from the use of less-approximated approaches. In this work, we chose a bead scheme based on Martini force field^22^ (with beads containing ∼4 non-hydrogen atoms), which was previously shown to represent an optimal compromise between efficiency and accuracy, allowing scattering calculations 50 times faster than the atomistic ones and with good accuracy for the range of interest of SAXS profiles.^50^ Importantly, form factors for Martini beads are available and were previously implemented in the PLUMED-ISDB module.^19,42,50^

Recently, we have implemented a hybrid multiresolution strategy to perform full atomistic MD simulations in which SAXS intensities, computed at a coarse-grain level based on the Martini force field, are used as restraints within the metainference framework^19^ (see Figure 1A). The virtual positions of the Martini beads are computed on-the-fly and are used in combination with Martini form factors^50^ for SAXS calculations. The computational efficiency of this strategy can be further improved using a multiple time-step protocol, where the metainference bias is applied only every few time steps.^51^ In our previous work, we demonstrated the reliability of the hybrid resolution approach for single-replica simulations in which two protein-nucleic acids complexes were refined against SAXS data. Here, we extended the described approach to multireplica M&M simulations, with the aim to exhaustively explore the conformational space of flexible biomolecules that are able to populate multiple conformational states.

### 2.3 Computational Details of the Simulations

K63-Ub_2_, for which both SAXS and PRE experimental data are available,^32,40^ was used as a test system. As a starting model for the simulations, we used the B and C chains of PDB 2ZNV:^37^ since the SAXS data we used to restrain the simulation were acquired on K63-Ub_2_ with distal-K63R mutation and proximal-D77 addition, we introduced these same modifications in our model. MD simulations were performed with GROMACS 2018,^52^ PLUMED 2,^43^ and the PLUMED-ISDB^42^ module, using the Amber ff03w force field^53^ with TIP4P/2005 water model^54^ and scaled protein–water Lennard-Jones parameters (amber03ws).^55^ The choice of this force field, which was specifically designed to increase molecules solvation, avoiding collapsed states and nonspecific protein–protein interactions, was guided by the fact that we expect an equilibrium between open and compact states of K63-Ub_2_ with only transient interdomain contacts. The system was solvated in a periodic dodecahedron box, initially 1.2 nm larger than the protein in each direction, and neutralized. After an initial energy minimization to a maximum force of 100 kJ/mol/nm, the solute was equilibrated under NVT conditions at a temperature of 300 K for 50 ps using the Berendsen thermostat;^56^ then, the Berendsen barostat was used to equilibrate the system in the NPT ensemble to a target pressure of 1 atm for 200 ps. The equilibration phase was followed by an initial MD simulation of 100 ns, from which a pool of well-equilibrated conformations was extracted to be used as starting models for the subsequent runs. During the production runs in the NPT ensemble, the MD integrator was employed with a time step of 2 fs; the temperature was maintained at 300 K using the Bussi thermostat,^57^ and the pressure was controlled with Parrinello–Rahman barostat.^58^ Bonds were constrained with the LINCS algorithm,^59^ using a matrix expansion of order 6 and 2 iterations per step. The electrostatic interaction was treated by using the particle mesh Ewald scheme^60^ with a short-range cutoff of 0.9 nm and a Fourier grid spacing of 0.12 nm; the van der Waals interaction cutoff was set to 0.9 nm.

Two metadynamics multireplica simulations were performed: (1) a metainference simulation, consisting of 32 replicas, in which metainference was used to enforce the agreement with SAXS data according to the hybrid approach (hySAXS); and (2) an unrestrained simulation, consisting of 8 replicas, in which similar settings of simulation (1) were used but without the inclusion of experimental restraints. PBMetaD was performed in combination with well-tempered metadynamics^61^ and the multiple-walker scheme,^49^ where Gaussians with an initial height of 1.0 kJ/mol were deposited every 0.4 ps, using a bias factor of 30. Four CVs were biased: two of them (hydContacts and polContacts) count the number of hydrophobic and polar contacts between the two ubiquitin domains, and the other two (TICAcv1 and TICAcv2) are the results of the linear combination of numerous angles as determined by a Time-lagged Independent Component Analysis^62^ (TICA) performed on the initial 100 ns MD simulation (see the Supporting Information for more details). The width of the Gaussians was determined with the dynamically adapted Gaussian approach,^63^ using a time window of 4 ps to estimate CVs fluctuations and setting as minimum values for the widths 0.01, 0.05, 0.01, and 0.01 for hydContacts, polContacts, TICAcv1, and TICAcv2, respectively.

Experimental SAXS intensities for K63-Ub_2_ are available in the SASDCG7^40^ entry of the SASDB database.^64^ For the hySAXS simulation, a set of 11 representative SAXS intensities at different scattering vectors, ranging between 0.06 Å^–1^ and 0.16 Å^–1^ and equally spaced, were included as restraints. The range of scattering vectors was selected based on the quality of the data, focusing on the less-noisy region of the experimental curve. We also note that the approximations of the SAXS forward model (i.e., the coarse-grain representation and the lack of hydration layer in the calculations) are more severe if wider angles also are considered, thus setting an upper limit to the possible range to be considered. These representative intensities were extracted from the experimental data, where a 21-point running average was performed to reduce the influence of experimental noise. Metainference was applied every 10 steps, using a single Gaussian noise per data point and sampling a scaling factor between experimental and calculated SAXS intensities with a flat prior between 0.5 and 1.5.

For the hySAXS simulation, each replica was evolved for 250 ns, resulting in a total simulation time of 8 μs; for the unrestrained simulation, 750 ns per replica were run, for a total of 6 μs. Convergence was assessed using the block analysis procedure, in which free-energy profiles are computed over different blocks of simulations and last, the weighted average error along the free-energy profile is computed as a function of the block length. In Figure S1 in the Supporting Information, the free-energy profiles and the block average analysis are reported, showing that both simulations converged with comparable errors. As a preliminary control, we checked the root-mean-square deviation (RMSD) of the single Ub domains. In both simulations, the Ub domains are well-folded. The comparison of RMSD distribution in the two simulations (Figure S2 in the Supporting Information) showed lower RMSD values for the hySAXS ensemble, with respect to the unrestrained one: that could be due to the shorter simulation time per replica, as well as a protective effect of the SAXS restraints against some destabilization resulting from the use of amber03ws.

### 2.4 Back-Calculation of PRE

In order to back-calculate PRE values from the obtained conformational ensembles, we used the following formula:^65^
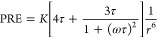
where τ is the correlation time, ω/(2π) the proton Larmor frequency, *K* a value that is dependent on the electron g-factor, the proton gyromagnetic ratio, and the magnetic moment of the free electron. Finally, *r* indicates the distance between the paramagnetic center and the nuclei. In the back-calculation, these distances were approximated with the distances between the Cβ atom of N25 or K48 and all of the amide hydrogens of the proximal ubiquitin. To account for this approximation, we evaluated an error of ±3 Å on the estimation of these distances, which finally gave us an estimation of the minimum/maximum PRE values.

## 3 Results and Discussion

To evaluate our hySAXS approach, after assessing its computational performances in comparison with conventional MD simulations and atomistic SAXS restraints, we tested its ability to improve the agreement of MD with experimental SAXS data in comparison with a state-of-the-art force-field (unrestrained simulations). As a model system, we employed K63-Ub_2_, for which independent data are available to validate our results.

### 3.1 The hySAXS Approach Is Computationally Efficient

In Figure 1B, we compared the performances of (i) a conventional atomistic MD simulation (yellow); (ii) all-atom metainference simulations, where SAXS restraints with atomistic forward model were included every step (green); and (iii) all-atom hySAXS simulations, where SAXS restraints were included every step (purple) or every 10 steps (blue). The use of the hybrid approach significantly improved the performances of SAXS-driven MD simulations, compared to the ones adopting atomistic scattering evaluation. This gain can be further increased using a multiple time-step protocol (Figure 1B, blue line), in which the restraint is applied every few time steps. This strategy is well justified in the case of SAXS data, which are characterized by slow temporal fluctuations, and allows one to approach the performances of conventional MD simulations.

### 3.2 Monitoring hySAXS Simulation

**Figure 2:**
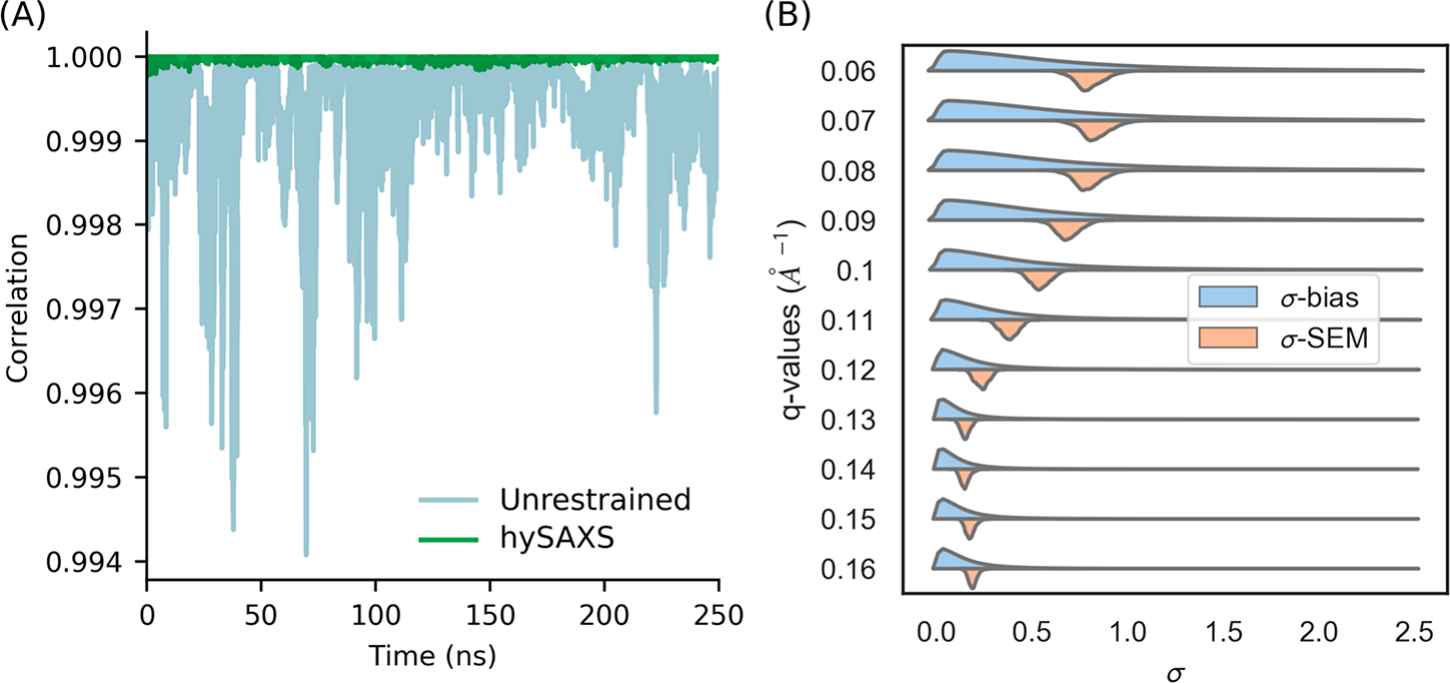
(A) Correlation, as a function of the simulation time, between experimental and back-calculated SAXS intensities, averaged over the replicas. The intensities considered are the ones used as restraints in the hySAXS simulation. (B) Probability density functions of the uncertainty parameters σ^Bias^ and σ^SEM^ (expressed in a.u.) for the 11 scattering angles considered.

To evaluate, on the fly, the effectiveness of SAXS restraints, we monitored the correlation between back-calculated and experimental data, as a function of the simulation time (Figure 2A), comparing hySAXS to an unrestrained simulation. The comparison revealed a better agreement in the hySAXS simulation (Figure 2A), confirming the efficacy of the restraints. This is supported by other statistical properties, including the sum of square deviation and the slope/intercept of the linear fit (see Figure S3 in the Supporting Information).

We also monitored the intensity of experimental restraints, which depends on the square sum of the uncertainty parameters σ_*r*,*i*_^Bias^ and σ_*r*,*i*_^SEM^ (cf. see earlier section entitled Theory and Methods). To this aim, we computed the distribution across the hySAXS ensemble of both σ_*r*,*i*_^Bias^, which is associated with experimental and forward model inaccuracies, and σ_*r*,*i*_^SEM^, i.e., the standard error of the mean over the replicas. We observed a broader distribution of the sampled parameter σ_*r*,*i*_^Bias^, with respect to σ_*r*,*i*_^SEM^ (Figure 2B), with greater uncertainties associated with smaller scattering angles (where, indeed, the global conformation primarily influences SAXS profiles). The values of σ_*r*,*i*_^SEM^ are always within the range sampled by σ_*r*,*i*_^Bias^, indicating that the two sources of error comparably contribute to the restraint weight and suggesting that the number of replicas (which concurs in determining the magnitude of σ_*r*,*i*_^SEM^) is sufficient.

### 3.3 Comparison of the Resulting Conformational Ensembles

**Figure 3:**
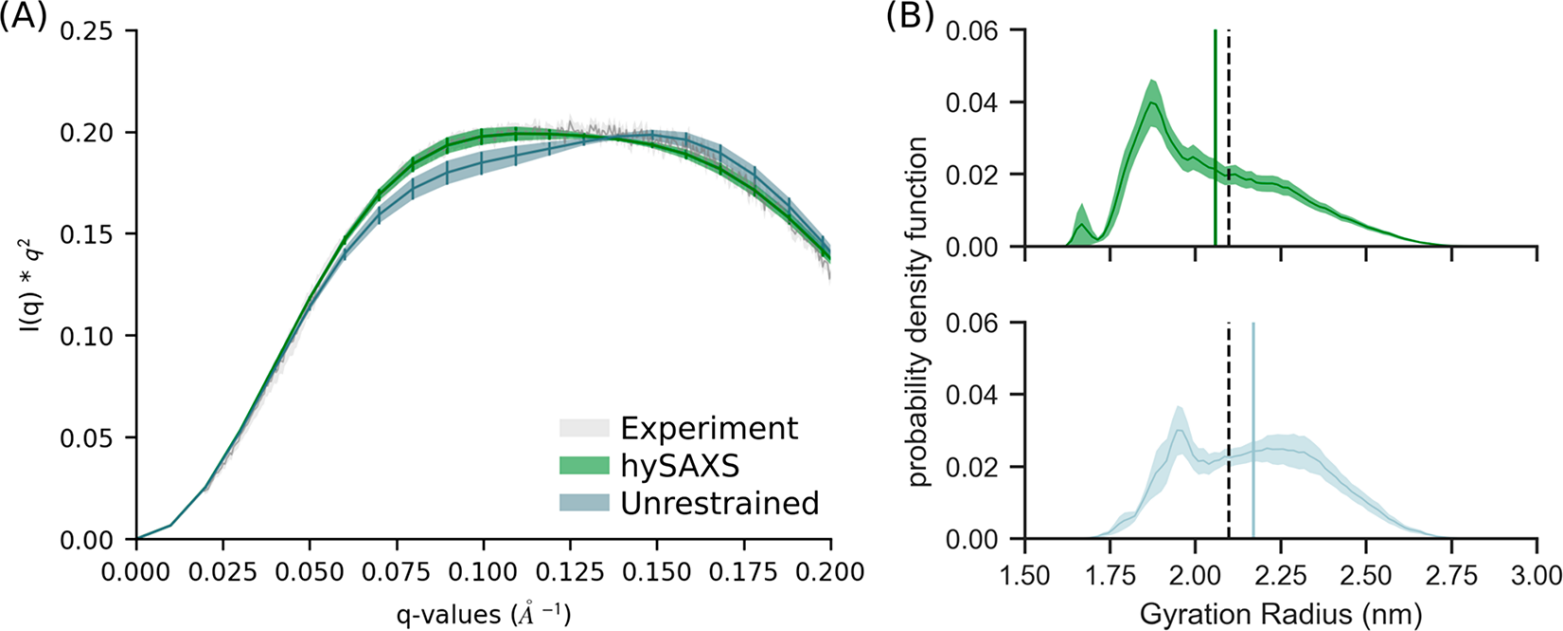
(A) Kratky plot comparing the experimental curve with the ones calculated (via atomistic approach) from the hySAXS and the unrestrained conformational ensembles. (B) Distribution of the gyration radius in the hySAXS (green) and in the unrestrained (light blue) conformational ensembles. Gyration radius were calculated using GROMACS. The vertical bars indicate the average back-calculated gyration radius, the shaded area indicates the standard error, computed via block-average analysis. The vertical black dashed bar indicates the SAXS-derived gyration radius.

The agreement with experimental SAXS data was eventually evaluated considering the entire conformational ensembles sampled within the unrestrained or hySAXS simulations. To this aim, we needed to estimate a scaling factor λ that relates experimental and calculated data. This value could, in principle, be determined by comparing the intensities at the *q* = 0 scattering angle, but since *I*(0) cannot be measured in SAXS experiments, we chose the λ that minimizes the χ^2^ (computed over 19 *q*-values in the range of 0.02–0.20 Å^–1^) between hySAXS and experimental intensities. We observed that hySAXS provides a better match with experimental data (as confirmed by the χ^2^ values: 0.44 and 3.6 for the hySAXS and unrestrained simulations, respectively), while the unrestrained ensemble strongly deviates from the experimental profile, showing a shape that is indicative of an oversampling of extended conformation (see Figure 3A, as well as Figure S4 in the Supporting Information). Importantly, our validation is conducted over a range of scattering angles (0.02–0.20 Å^–1^) larger than the one used for restraining the simulation, and our conclusions are not dependent on the choice of the scaling factor: indeed, hySAXS simulation provides a better agreement with experiments also when choosing a λ value that minimizes the χ^2^ value of unrestrained intensities (see Figure S5 in the Supporting Information).

Accordingly, we noticed a remarkable effect of SAXS restraints on the interdomain dynamics, as shown by the comparison of the probability density function of the gyration radius and the minimum interdomains distance (see Figure 3B, as well as Figure S6 in the Supporting Information). Both of the ensembles populate a wide range of gyration radius values (spanning from 1.5 nm to 3.0 nm), in agreement with the observation that K63-Ub_2_ exists in a dynamic ensemble comprising both extended and compact states. Nevertheless, the hySAXS ensemble prefers more-compact conformations, resulting in an average gyration radius of 2.06 ± 0.03 nm, in contrast with that obtained for the unrestrained ensemble (2.17 ± 0.05 nm). The value obtained from the hySAXS ensemble better approaches the SAXS-derived gyration radius (2.10 ± 0.01 nm), with a small difference that could be explained by the contribution of the hydration layer.^67^ Altogether, our results support the idea that, while the hySAXS ensemble better reproduces the correct balance between compact and open states, the unrestrained ensemble overestimates the population of extended conformations. Importantly, here, we showed that SAXS restraints could be effectively used to contain this trend.

The propensity toward extended states for the unrestrained ensemble could be explained by the use of the amber03ws, which is a force field that was specifically designed to prevent the overstabilization of IDPs compact states by strengthening water–protein interaction terms. Our results, which are consistent with a previous report,^68^ suggest that this modification could be too strong for folded and multidomain proteins, leading to the destabilization of compact conformations. Recent huge efforts in force-field development indicate that refinement of few force-field terms (as torsional parameters or water models), while useful in improving the description of either well-folded proteins or IDPs, could be not sufficient to provide an equally accurate description of both.^68,69^

**Figure 4:**
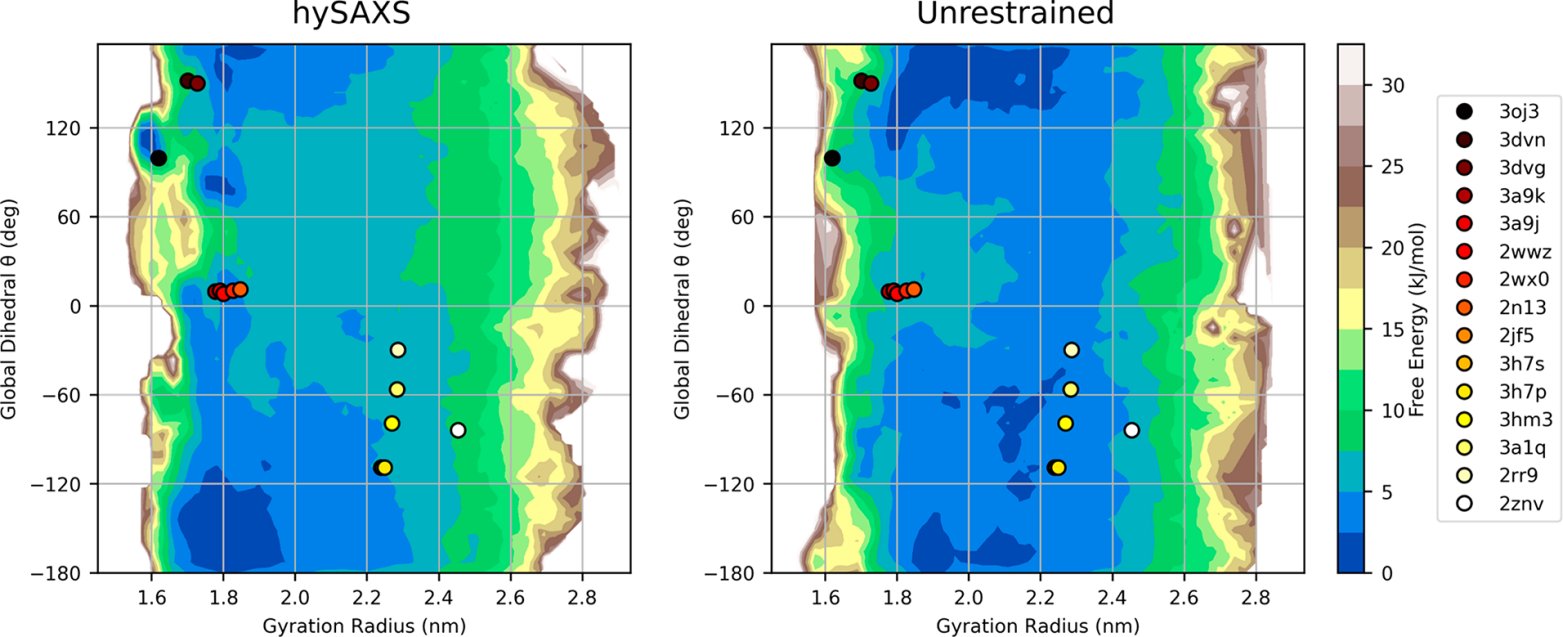
Two-dimensional free-energy surface for K63-Ub_2_, derived by the hySAXS (left panel) and the unrestrained (right panel) ensembles, as a function of the Cα-gyration radius and the global dihedral angle θ (see section S1 and Figure S7 in the Supporting Information). The coordinates of the available PDB structures in this space are plotted with points (colored from red to white, from more compact to extended conformations). To make the gyration radius comparable between simulations and PDB structures, only Cα atoms of residues 1–72 of the two ubiquitin domains were considered for gyration radius calculations.

Lastly, in Figure 4, we reconstructed two-dimensional free-energy landscapes in a space defined by the Cα-gyration radius and a global dihedral angle θ (also used as metadynamics CVs; see Figure S7 in the Supporting Information) that describes the relative orientation of the two ubiquitin domains. Interestingly, the coordinates in this space of the available K63-Ub_2_ PDB structures mostly fall in regions characterized by low free energy, according to both hySAXS and unrestrained simulations, indirectly supporting the reliability of the employed force field. The inspection of the free energies revealed that, in the two ensembles, the Ub domains can reorient freely when extended but prefer different Ub–Ub orientation in compact conformations. The contact map analysis (see Figure S8 in the Supporting Information) confirms the absence of highly stable interdomains contacts, supporting the idea that numerous different interfaces are accessible, and it shows that, in the two simulations, diverse groups of residues are preferred for interdomain interactions, where the major differences concern the residues of distal ubiquitin (the majority of contacts are engaged by distal residues 42–49, according to hySAXS and by residues 6–11, according to the unrestrained simulation). Based on this observation, we hypothesized that SAXS restraints could help in sampling more-reliable protein/protein interfaces. To test this hypothesis, we proceeded by validating our conformational ensemble against PRE data.

### 3.4 Validation and Analysis of the Ub/Ub Interfaces

PRE experiments from NMR are particularly suited to provide information about intersubunit distances in multidomain proteins. In these experiments, after conjugation of a specific residue with a paramagnetic probe, PRE can be measured for the other domain, where PRE values are proportional to the inverse sixth power of the distance between the paramagnetic center and the nuclei. Becuase of this functional form, PRE data are extremely sensitive to closed states even if sparsely populated.^65^ Therefore, a comparison of the conformational ensemble against PRE is particularly indicated to validate the Ub/Ub interfaces of the compact states and their relative population.

Liu and co-workers previously acquired intersubunit PRE data for K63-Ub_2_, conjugating the paramagnetic probe on residues N25 or K48 of the distal ubiquitin, after N25C/K48C mutations, and detecting many large PRE for some residues of the proximal unit.^32,70^ We back-calculated the same PRE values from our hySAXS and unrestrained conformational ensembles (see the Theory and Methods section) and compared them with those determined from experiments.

**Figure 5:**
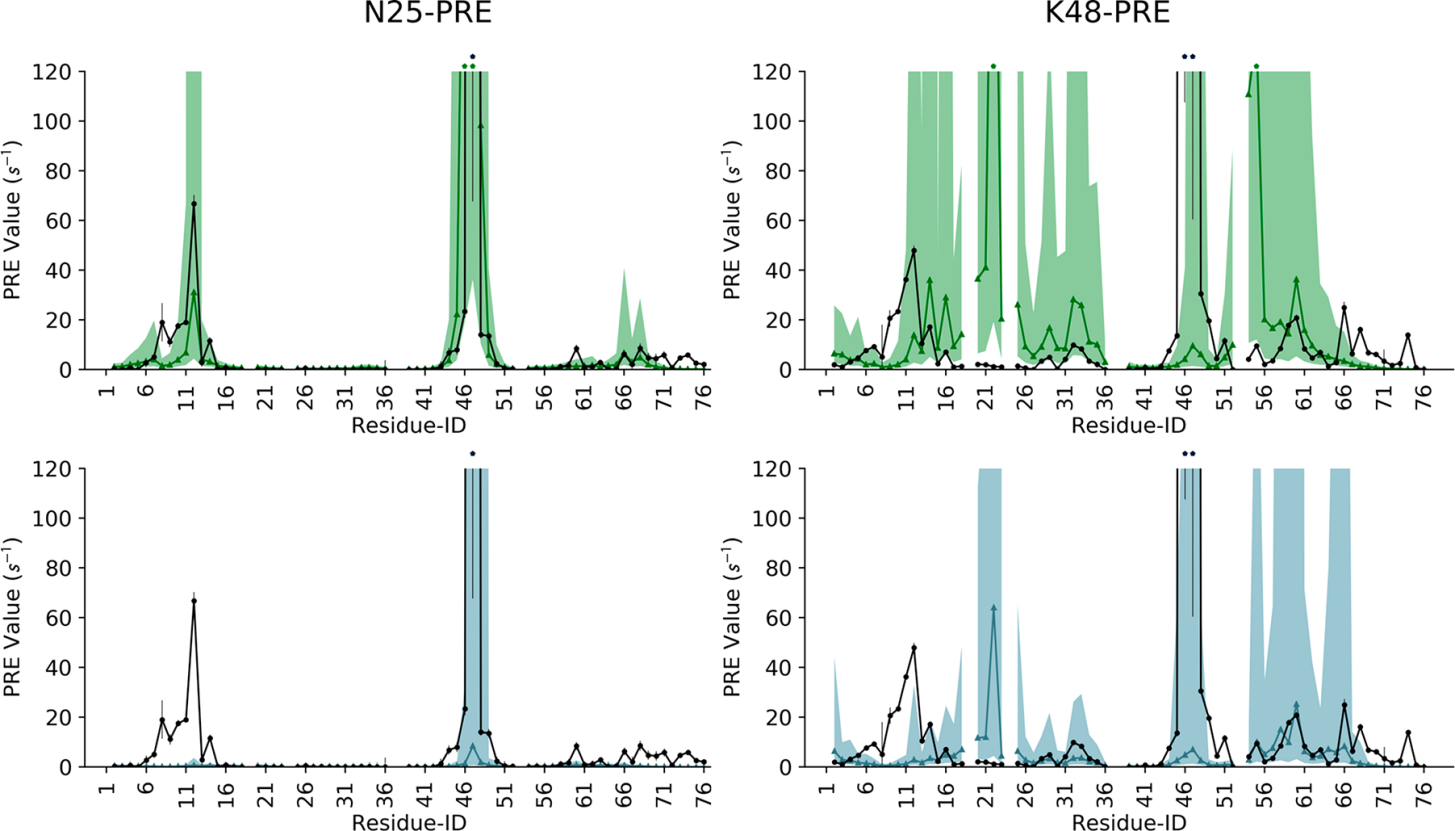
Comparison of experimental (black line) and back-calculated intersubunit PRE for the residues of K63-Ub_2_ proximal ubiquitin, with the paramagnetic probe conjugated at N25C (left panels) or K48C (right panels) of the distal ubiquitin. The area between the minimum/maximum back-calculated PRE values, considering a ±3 Å error on the estimation of probe–nuclei distances, is shaded green or light blue, for hySAXS and unrestrained ensembles, respectively. The respective back-calculated PRE, without distance correction, is shown with green and light-blue lines. PRE values of >120 s^–1^ are indicated with an asterisk on the top of the graph.

We observed that experimental N25-PRE is in good agreement with those calculated from the hySAXS ensemble (Figure 5, upper-left panel), suggesting that the compact interfaces are correctly sampled in our hySAXS run. Conversely, the unrestrained ensemble fails to reproduce N25-PRE for the proximal unit residues 8–14 (Figure 5, lower-left panel). Both the hySAXS and the unrestrained ensembles correctly identify the regions where high K48-PRE are detected (Figure 5, right panels); nevertheless, in both cases, we observe a significant overestimation of the PRE involving the residues 20–23 of the proximal unit.

**Figure 6:**
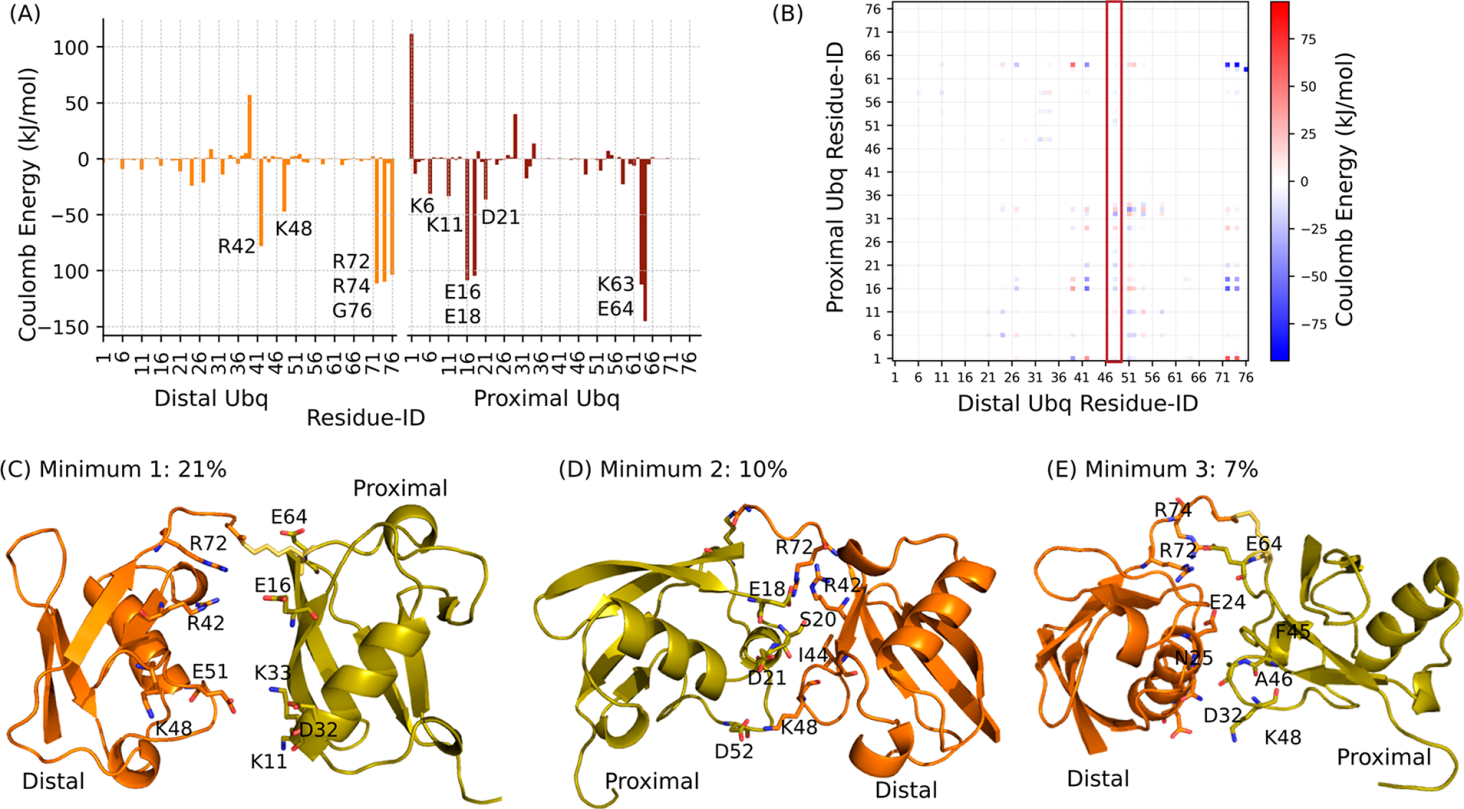
(A) Per-residue Coulomb energy obtained summing over the residue–residue energetic contributions for pairs of residues belonging to the two different Ub domains. Residues of distal and proximal ubiquitin are colored in orange and red, respectively; the lowest energy peaks are labeled. (B) Coulomb energy matrix reporting on the electrostatic interactions between the two domains. The column corresponding to the interactions engaged by distal K48 is highlighted in red. (C–E) Representative conformations have been extracted from the main minima of the compact state. Their population is reported and relevant residues for the interface are highlighted in sticks. Figures were created with Pymol software (The PyMOL Molecular Graphics System, Version 2.0 Schrödinger, LLC).

Since the comparison with both N25 and K48-PRE supports the reliability of our hySAXS ensemble in sampling correct Ub/Ub interfaces, we hypothesized that the observed deviations could arise as a consequence of the introduction of the paramagnetic probe at the K48 site in PRE experiments, along with the K48C mutation. Indeed, while N25C mutation is more conservative, the replacement of a charged amino acid (K48C) could destabilize relevant interdomains contacts. To support this hypothesis, we analyzed the energetic contributions of each residue to the interface formation. We found that, according to the hySAXS ensemble, K48 of proximal ubiquitin is important in stabilizing electrostatic interactions at the interface and that a part of these contacts are indeed engaged with the negatively charged D21 residue of distal ubiquitin, belonging to the region where the major deviations were observed (see Figures 6A and 6B). Importantly, we verified that this is not the case for N25, where neither Coulomb nor Lennard-Jones interactions seem to play a major role in stabilizing the Ub/Ub interfaces (see Figure 6A, as well as Figure S9 in the Supporting Information).

In order to have a deeper insight into the sampled Ub/Ub interfaces, we analyzed the conformational minima identified by our hySAXS run. The pool of compact conformations (defined as the ones with a Cα-gyration radius of <2.0 nm and accounting for 57% of the conformational space) were clustered, based on the backbone RMSD with a cutoff of 6 Å. This procedure identified three main clusters, with populations of 21%, 10%, and 7%, respectively. As expected, these three conformational minima contain very heterogeneous conformational states (see Figure S10 in the Supporting Information), supporting the idea that K63-Ub2 can transiently populate many different possible interfaces. Nevertheless, the inspection of both their structures and of the corresponding energy matrices (Figure S10) allowed us to characterize the interfaces and the contacts driving the interdomain recognition in greater detail (see Figures 6C–E). We observed that, in all three minima, the positive residues R42, R72, and/or R74 of distal ubiquitin engage electrostatic interactions with negatively charged residues of the proximal domain (mainly E16–E64, E18, and E64 for minima 1, 2, and 3, respectively). In addition to these interactions, further contacts characterize the different minima, again involving mainly charged residues (Figures 6C–E). While, in the most populated minimum (minimum 1), hydrophobic interactions are almost absent, these are present in the other minima: in minimum 2, contacts between distal I44 and proximal S20 are observed, whereas, in minimum 3, the interface is also stabilized by contacts between the aliphatic side chains of distal residues E24–N25 and the proximal F45–A46.

Overall, our analysis revealed the involvement of many charged residues in the Ub/Ub interface and suggests that K63-Ub_2_ prefer electrostatic interfacial contacts, being hindered by steric constraints to interact via the common I44/I36 hydrophobic patches, which is consistent with previous reports.^71^ Our results are in agreement with previous mutagenesis experiments concerning the E64 residue of the proximal unit, which plays a major role in both minima 1 and 3 interfaces. Indeed, it was reported that E64 is important for the stabilization of closed conformations, where an E64R mutation was shown to decrease the binding affinity toward ligands, known to bind the K63-Ub_2_ closed states, via an entropically driven mechanism. Herein, our results support the conformational selection mechanism proposed by Liu and co-workers^32^ for K63-Ub_2_ ligand recognition.

## 4 Conclusions

In this work, we have presented a hybrid-resolution MD-based strategy, which is useful with regard to determining conformational ensembles that provide an accurate interpretation of SAXS data. The proposed approach makes the inclusion of SAXS in MD simulations feasible, in terms of computational efficiency, without losing atomistic details, and allows us to deal with highly flexible systems, aiding in the estimation of the population of the different existing conformational states.

To prove the efficacy of the method, here, it has been applied to study the conformational ensemble of the multidomain protein K63-Ub_2_. Our results reveal that the inclusion of SAXS restraints can significantly influence the relative positioning of the different subunits and the degree of protein extension, improving the reliability of the conformational sampling, as supported by indirect validations and by quantitative comparison with independent experimental data.

## Supporting Information Available

Description of metadynamics Collective Variables (section S1). Figures: 1. Simulation convergence (Figure S1); RMSD pdf of the single ubiquitin domains (Figure S2); statistical parameters to monitor hySAXS simulation (Figure S3); SAXS curves with residual plot (Figure S4); Kratky plot (Figure S5); interdomain minimum distance probability distribution (Figure S6); graphical representation of the global dihedral angle θ (Figure S7); contact map of the hySAXS and unrestrained ensemble (Figure S8); per-residue Lennard-Jones interface contribution (Figure S9); and energy matrices and conformational ensembles for the hySAXS compact minima (Figure S10) (PDF)

## ABBREVIATIONS

K63-Ub_2_: K63-linked diubiquitin
MD: molecular dynamics
M&M: metadynamics metainference
PBMetaD: parallel bias metadynamics
CV: collective variable
SAXS: small-angle X-ray scattering
PRE: paramagnetic relaxation enhancement
NMR: nuclear magnetic resonance
FRET: Förster resonance energy transfer
TICA: time-lagged independent component analysis
