# Supplementary material for: Determination of Protein Structural Ensembles by Hybrid-Resolution SAXS Restrained Molecular Dynamics
Source: J Chem Theory Comput. 2020 Mar 2;16(4):2825–34. doi: 10.1021/acs.jctc.9b01181 (PMC7997378; doi:10.1021/acs.jctc.9b01181)
Supplement: Supplementary file 1 — ct9b01181_si_001.pdf [file ct9b01181_si_001.pdf]

Supplementary Information

Determination of protein structural ensembles by hybrid-resolution SAXS restrained Molecular Dynamics.

Cristina Paissoni[1,*], *Alexander Jussupow*[2], *and Carlo Camilloni*[1,*]

[1]Dipartimento di Bioscienze, Università degli Studi di Milano, via Celoria 26, 20133 Milano, Italy [2]Department of Chemistry and Institute of Advanced Study, Technical University of Munich, Garching 85747, Germany.

S1. Metadynamics Collective Variables

Four variables were used as CVs in PBMetaD: two of them (hydContacts and polContacts) count the number of hydrophobic and polar contacts between the ubiquitin domains, the other two are the results of a Time-lagged Independent Component Analysis[1] (TICA) performed on the initial 100 ns MD simulation.

The form of the variables hydContacts and polContacts is:

$$CV = \sum_{i \in A} \sum_{j \in B} \frac{1 - (\frac{r_{ij}}{r_0})^N}{1 - (\frac{r_{ij}}{r_0})^M},$$

where r_{ij} is the distance between the atoms i and j, belonging to group A and B, respectively, $r_0 =$ 5Å, N=6, M=10 for hydContacts and M=12 for polContacts. In hydContacts, the groups A and B are defined as the collection of the hydrophobic amino-acids side-chains carbons for distal and proximal ubiquitin; in polContacts, they are defined as the collection of the donor/acceptor atoms belonging to distal and proximal ubiquitin.

The form of the two TICA CVs is:

$$CV = \frac{1}{2} \sum_i K_i \left[1 + \cos\left(\alpha_i - \alpha_i^{ref}\right)\right],$$

where the sum runs over a certain number of torsions of interest, α_i is the instantaneous value of the torsion, α_i^{ref} is a user specified reference value and K_i are coefficients. While the same set of torsions is used in TICAcv1 and TICAcv2, the K_i coefficients are different in the two CVs and they were determined following the TICA procedure described in reference [1] using the PyEMMA tool[2]. Initially, K_i coefficients were set to be all equal and normalized to 1; from the 100 ns MD

simulation, with the help of the *pyemma.coordinates* package, we computed the mean-free covariance and time-lagged covariance matrix to obtain eigenvalues, eigenvectors and implied time-scales. We choose a lag time of τ =6 ns, in a regime where the eigenvectors coefficients are stable. Since the results show the presence of two dominant eigenvalues, we selected as new CVs the two associated eigenvectors, providing the two sets of coefficients K_i. The torsions considered include the dihedral angles of the flexible region connecting the two ubiquitin domains (i.e. the *phi/psi* angles of the residues 72-76 in the distal ubiquitin and the *chi* dihedral angles of K63 in proximal ubiquitin) plus three global (dihedral) angles, denoted as θ, γ and α. In order to define θ, γ and α we used the position of the Cα atoms center for the following groups of residues:

1. G1: residues 24 to 32, corresponding to alpha helix 1;
2. G2: residues 42 to 45 and 68 to 71, corresponding to beta strands 3 and 5;
3. G3: residues 3 to 6 and 13 to 16, corresponding to beta strands 1 and 2;
4. G4: all the residues of groups 1-3 (i.e. residues 3-6, 13-16, 24-32, 42-45, 68-71)
5. G5: residues 71 to 76 of the distal ubiquitin and residue 63 of the proximal ubiquitin, corresponding to the linker region connecting the two ubiquitin domains.

The global angles are then defined as:

1. θ: G1(Prox)-G2(Prox)-G2(Dist)-G1(Prox), using a reference value α_i^{ref}= 1.2 radiant;
2. γ: G2(Prox)-G3(Prox)-G1(Dist)-G2(Prox), using a reference value α_i^{ref}= 1.2 radiant;
3. α: G4(Prox)-G5-G4(Dist), using a reference value α_i^{ref}= 0 radiant.

Here Prox and Dist indicate proximal and distal ubiquitin, respectively.

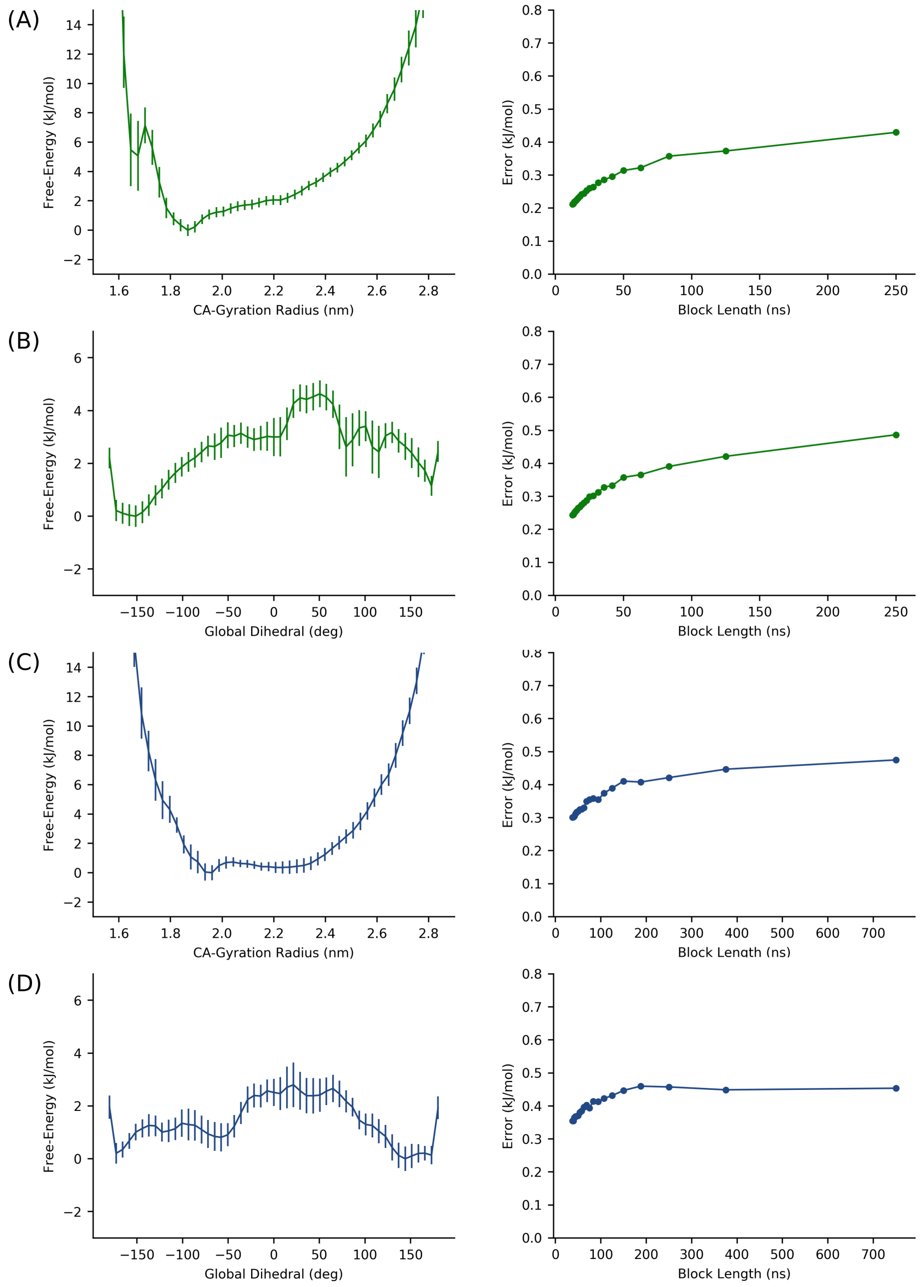

Figure S1. Free-energy profiles (left panel) and block-average analysis (right panel) for hySAXS (A and B) and unrestrained (C and D) simulations. The free-energy error bars indicate the standard error computed via block-average analysis. Block lengths were chosen to be a fraction (1/20 to 1) of the length of the replica. The variables considered are the Cα-gyration radius and the global dihedral angle θ, later used to reconstruct 2d-free energy landscapes.

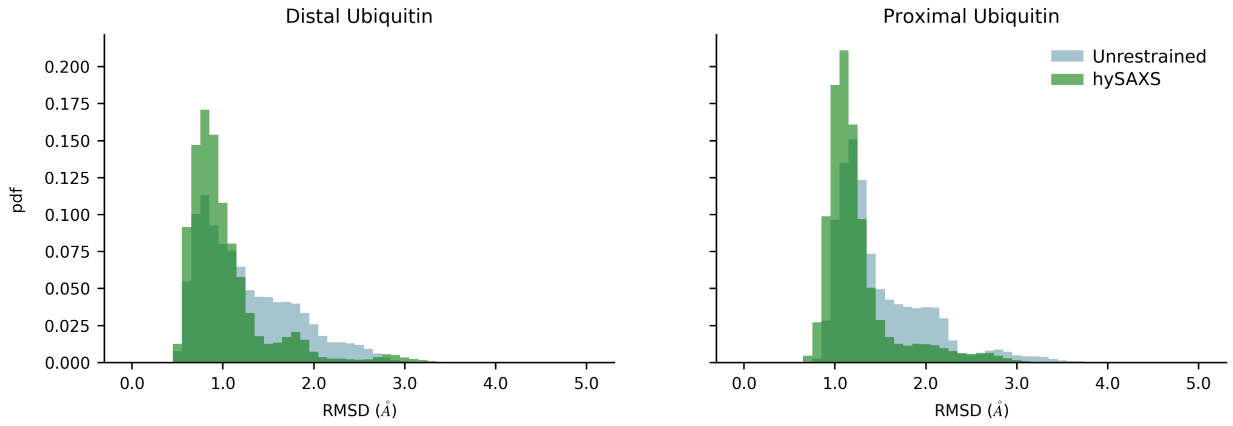

Figure S2. RMSD probability density functions of the single ubiquitin domains with respect to a reference crystallographic structure (PDB: 1UBQ), computed for both the hySAXS (green) and the unrestrained (light blue) ensembles. The RMSD was computed over the backbone atoms of residues 1-71 for each ubiquitin domain.

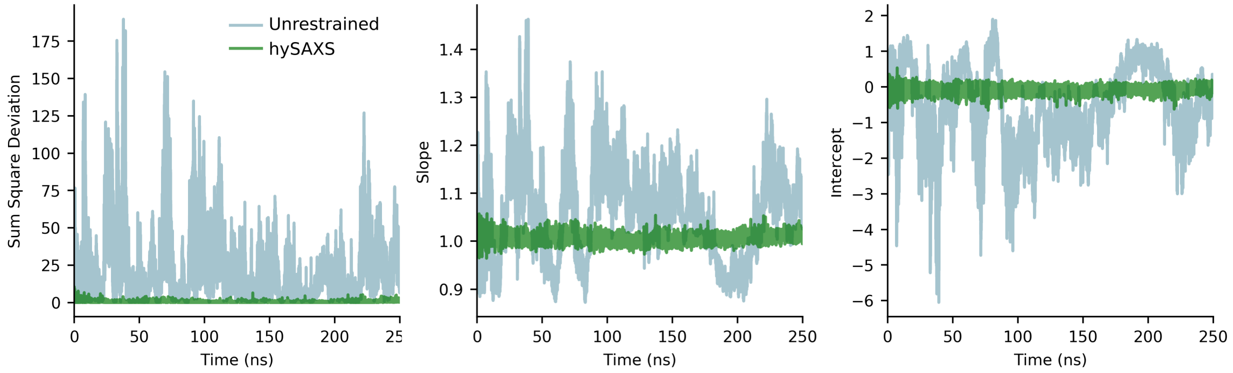

Figure S3. Different statistical properties (including the sum of square deviations, the slope and the intercept of a linear fit), as a function of the simulation time, reporting on the agreement between experimental and back-calculated SAXS intensities. The intensities considered are the ones used as restrained in the hySAXS simulation. The back-calculated data are averaged over the replicas and computed with the coarse-grained approach.

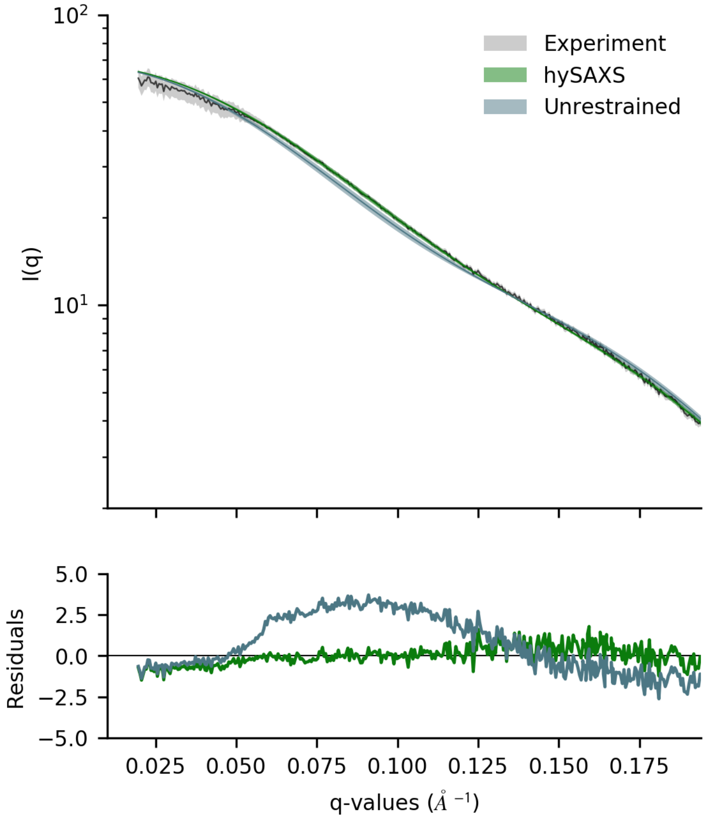

Figure S4. SAXS profiles and residual plot comparing the experimental curve (gray) with the ones back-calculated, via atomistic approach, from the hySAXS (green) and the unrestrained (light blue) conformational ensembles.

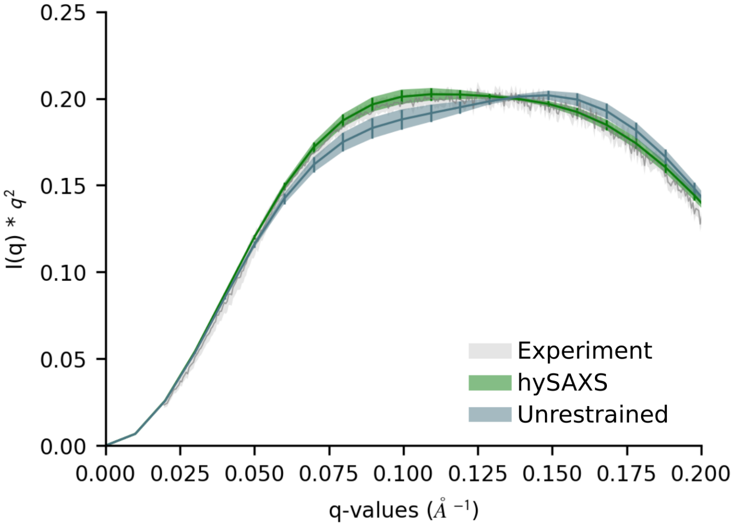

Figure S5. Kratky plot comparing the experimental curve with the ones calculated (via atomistic approach) from the hySAXS and the unrestrained conformational ensembles. The scaling factor λ was chosen to minimize the χ^2 of unrestrained intensities versus the experimental ones, considering 19 q-values in the range 0.02-0.20 Å^{-1}. The obtained χ^2 values are 1.2 for the hySAXS and 2.8 for the unrestrained ensemble.

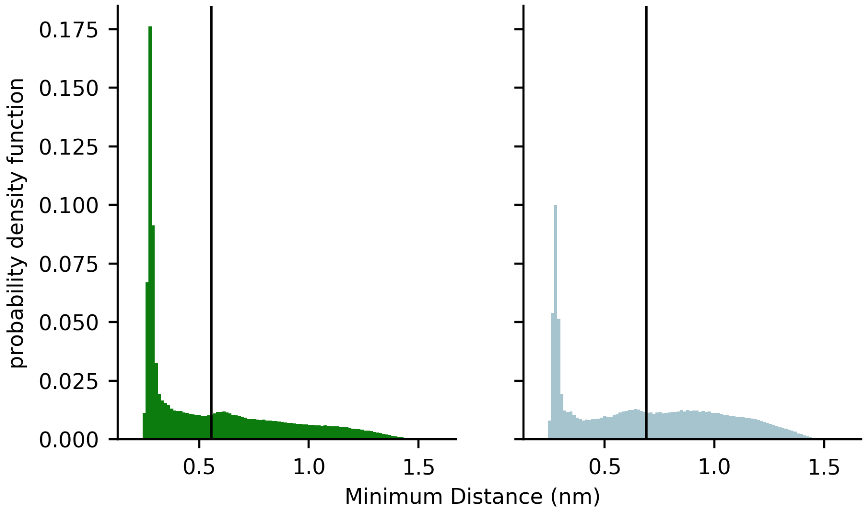

Figure S6. Distribution of the minimum distance between the two ubiquitin domains (considering only residues 1-72 for each subunit), according to the hySAXS (green) and the unrestrained (light blue) ensembles. The vertical bars indicate the respective averages.

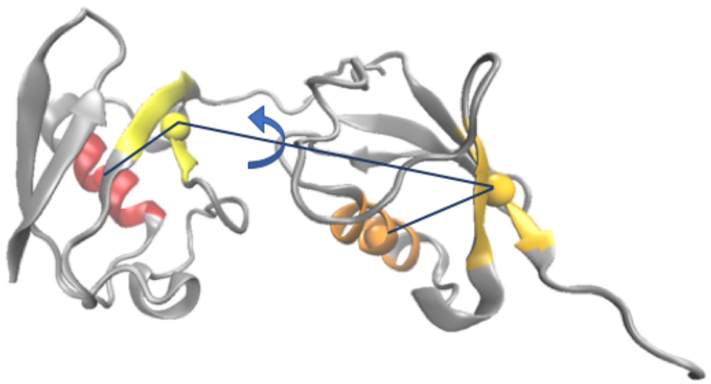

Figure S7. Graphical representation of the global dihedral angle θ, defined as the dihedral angle connecting the group of atoms: G1(Prox)-G2(Prox)-G2(Dist)-G1(Prox) (see also Section S1). The groups G1 and G2 are defined as the Cα atoms center of residues 24-32 (alpha helix 1) and residues 42-45/68-71 (beta strands 3 and 5), respectively.

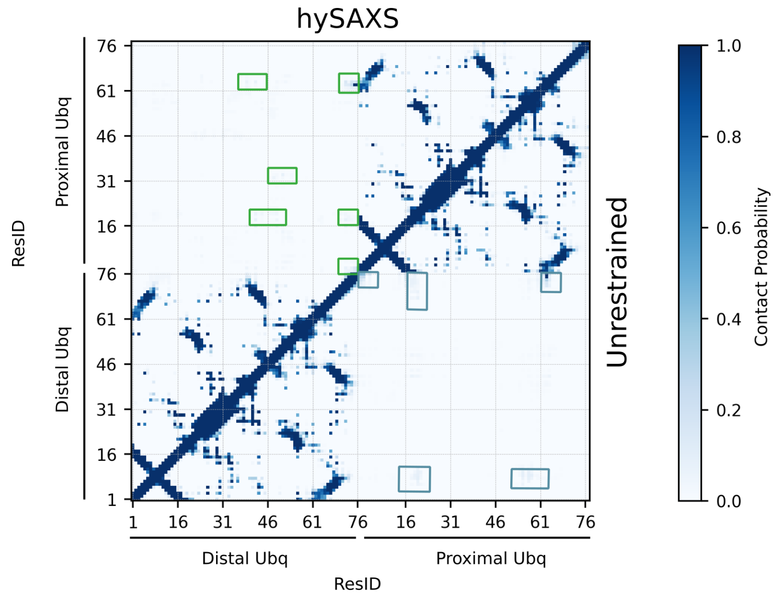

Figure S8. Contact maps for the hySAXS (upper left part of the matrix) and the unrestrained (lower right) ensembles. The color code corresponds to the probability of contact formation, where a contact is formed if at least a pair of non-hydrogen atoms, belonging to the two residues, is closer than 0.6 nm. Inter-domains contacts with relatively high probability (>5%) are highlighted with green and blue boxes for the hySAXS and the unrestrained ensembles, respectively.

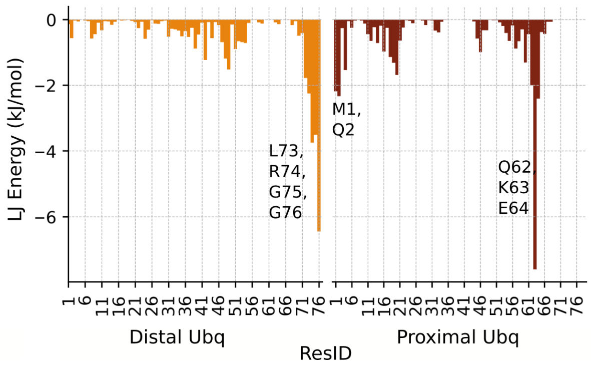

Figure S9. Per-residue Lennard-Jones energy obtained summing over the residue-residue energetic contributions for residues pairs belonging to the two different Ub domains. Residues of distal and proximal ubiquitin are colored in orange and red; the lowest energy peaks are labeled.

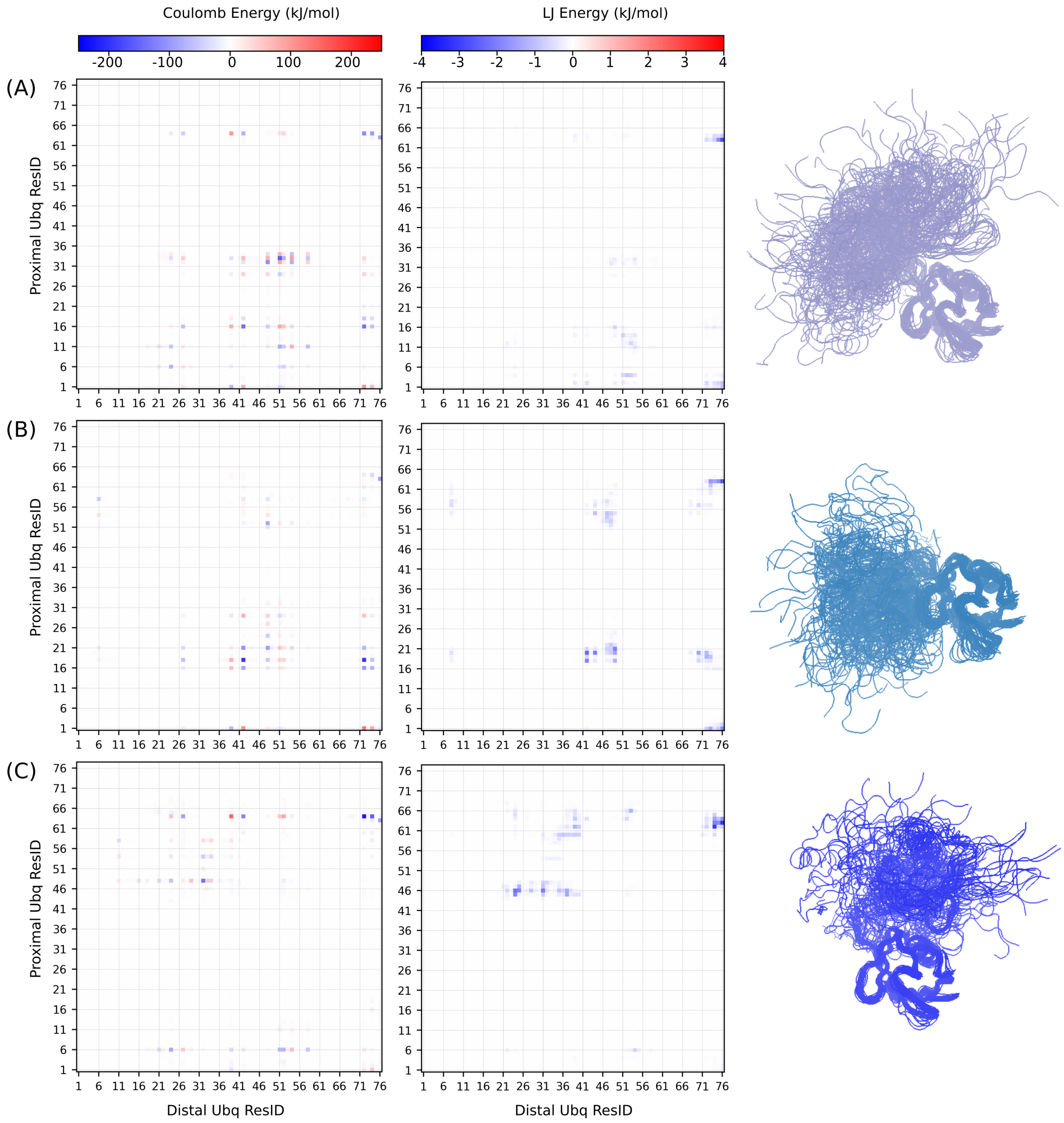

Figure S10. For minima 1, 2 and 3 are reported the Coulomb (left panel), the Lennard-Jones (central panel) energy matrices and the conformational ensemble (right panel).

REFERENCES

(1) McCarty, J.; Parrinello, M. A Variational Conformational Dynamics Approach to the Selection of Collective Variables in Metadynamics. *J. Chem. Phys.* **2017**, *147* (20), 204109. https://doi.org/10.1063/1.4998598.

(2) Scherer, M. K.; Trendelkamp-Schroer, B.; Paul, F.; Pérez-Hernández, G.; Hoffmann, M.; Plattner, N.; Wehmeyer, C.; Prinz, J. H.; Noé, F. PyEMMA 2: A Software Package for Estimation, Validation, and Analysis of Markov Models. *J. Chem. Theory Comput.* **2015**, *11* (11), 5525–5542. https://doi.org/10.1021/acs.jctc.5b00743.
